# Long noncoding RNAs regulating enzymatic reactions in cancer

**DOI:** 10.1038/s12276-025-01464-7

**Published:** 2025-08-14

**Authors:** Chang Hoon Shin, Kyungmin Kim, Chul Woong Ho, Ji Won Lee, Min Jeong Jo, Kyung-Won Min, Je-Hyun Yoon

**Affiliations:** 1 Department of Oncology Science, University of Oklahoma, Oklahoma City, OK USA; 2 Department of Biology, College of Natural Sciences, Gangneung-Wonju National University, Gangneung-si, Republic of Korea; 3 Department of Pathology, University of Oklahoma, Oklahoma City, OK USA

**Keywords:** Long non-coding RNAs, Ubiquitylation

## Abstract

Long noncoding RNAs (lncRNAs) have emerged as critical regulators of cancer development and progression by modulating enzymatic reactions involved in cellular metabolism and protein modifications. These noncoding transcripts exert their roles through molecular interactions with target proteins. Here we explore the diverse mechanisms through which lncRNAs optimize enzymatic reactions in metabolic processes and protein modifications, both of which are fundamental to the growth, adaptation, and survival of cancer cells. These processes, often dysregulated during cancer progression, are modulated by lncRNAs through direct interactions with specific proteins, underscoring their critical role in oncogenesis. Key research findings are summarized to provide a comprehensive understanding of the roles of lncRNAs in modulating these cellular processes. This Review offers an in-depth overview of the complex regulatory networks through which lncRNAs influence cancer pathogenesis and provides essential insights for the development of future therapeutic strategies.

## LncRNAs shape cancer metabolism and protein modifications

This Review explores how long noncoding RNAs (lncRNAs) play a role in cancer by modulating metabolism and protein modifications. LncRNAs are RNA molecules that do not code for proteins but can regulate various cellular processes. The study addresses the gap in understanding how lncRNAs influence cancer metabolism and posttranslational modifications (PTMs), which are changes made to proteins after they are formed. Researchers found that lncRNAs can bind to metabolic enzymes, altering their activity and affecting cancer initiation and development. For example, lncRNAs can enhance or inhibit glycolysis and the tricarboxylic acid cycle. They also regulate PTMs such as phosphorylation, ubiquitination, and SUMOylation. The study concludes that lncRNAs are crucial in fine-tuning these processes, offering potential targets for cancer therapy. Future research could further explore these interactions to develop new cancer treatments.

This summary was initially drafted using artificial intelligence, then revised and fact-checked by the author.

## Overview

Eukaryotic cells adapt to environmental changes by reprogramming cellular metabolism for optimal growth and survival while maintaining metabolic plasticity^1,2^. These cellular processes contribute to normal physiology as well as the progression of diseases, including cancer, neurodegeneration, and immune disorders^3–5^, highlighting the crucial role of metabolic regulation at the cellular, tissue, organ, and systemic levels^6^. Metabolic reprogramming, one of the hallmarks of cancer, enables tumor cells to modulate their metabolism in a way that affords beneficial dynamic bioenergetic alterations within the tumor microenvironment^7,8^. Metabolic adaptation influences the production of oncometabolites that contribute to cancer initiation and progression by regulating gene expression and the tumor microenvironment. This often involves altering cancer-related processes such as epigenetic regulation, signal transduction, and immune suppression^9–12^. Cancer-specific metabolism also renders these transformed cells resistant to various therapies, thereby reducing treatment efficacy and worsening patient prognosis^13,14^. Emerging evidence indicates that cancer cells adapt their metabolic flux in response to changes in nutrient availability by altering the expression or activity of metabolic enzymes. Through metabolic reprogramming, cancer cells exploit metabolic intermediates and byproducts to support their aggressive phenotypes^1,15^. Nevertheless, the precise molecular mechanisms regulating metabolic reprogramming in cancer remain elusive.

Understanding cancer-specific metabolic reprogramming in response to subtle changes in nutrients within the competitive tumor microenvironment is crucial for identifying potential therapeutic targets and strategies to inhibit tumor progression. The most well-established signaling pathways orchestrating metabolic reprogramming are the mechanistic target of rapamycin complex (mTOR) and AMP-activated protein kinase (AMPK) pathways, which respectively control anabolic and catabolic processes^16–18^. However, an understanding of these regulatory pathways alone may not provide a sufficiently comprehensive overview of how adaptive metabolic processes are fine-tuned. Thus, a more thorough characterization of how metabolic enzyme activities and expression are regulated by *trans*-acting factors will help to identify potential therapeutic targets for cancer treatment.

Another critical molecular process that is altered in magnitude during cancer development is posttranslational modifications (PTMs), including phosphorylation, ubiquitination, SUMOylation, glycosylation, acetylation, and methylation, all of which play significant roles in cancer^19–23^. PTMs regulate cellular homeostasis in cancer progression and survival by modulating protein states and coordinating with metabolic reprogramming. These modifications influence protein activity, stability, subcellular localization, and protein–protein interactions, enabling cancer cells to survive, proliferate, and metastasize^24^. PTMs form an intricate regulatory network that supports tumorigenesis by facilitating signaling pathways and cellular responses. However, how PTMs, alongside metabolic reprogramming, are precisely regulated to drive and sustain the malignant traits of cancer remains unclear.

Recent research has highlighted the reciprocal interactions between long noncoding RNAs (lncRNAs) and metabolic as well as nonmetabolic enzymes, underscoring the pivotal role of lncRNAs as novel contributors to cancer metabolic reprogramming and PTMs through their fine-tuning regulatory activity^25,26^. LncRNAs, which are over 200 nucleotides in length, account for a substantial portion of the transcriptome^27,28^. Their biogenesis and processing share many similarities with those of mRNAs, as both are transcribed by RNA polymerase II with a few exceptions. However, they differ in chromatin states, 5′-capping, 3′-polyadenylation, and nuclear export^29^. Generally, lncRNAs are shorter, tend to have fewer exons, and exhibit lower abundance than mRNAs^30,31^. LncRNAs regulate various cancer-related characteristics, including proliferation, survival, migration, invasion, metastasis and genomic stability^27^. Nevertheless, research elucidating the roles of lncRNAs in metabolic regulation and PTMs is still in its early stages.

**Fig. 1:**
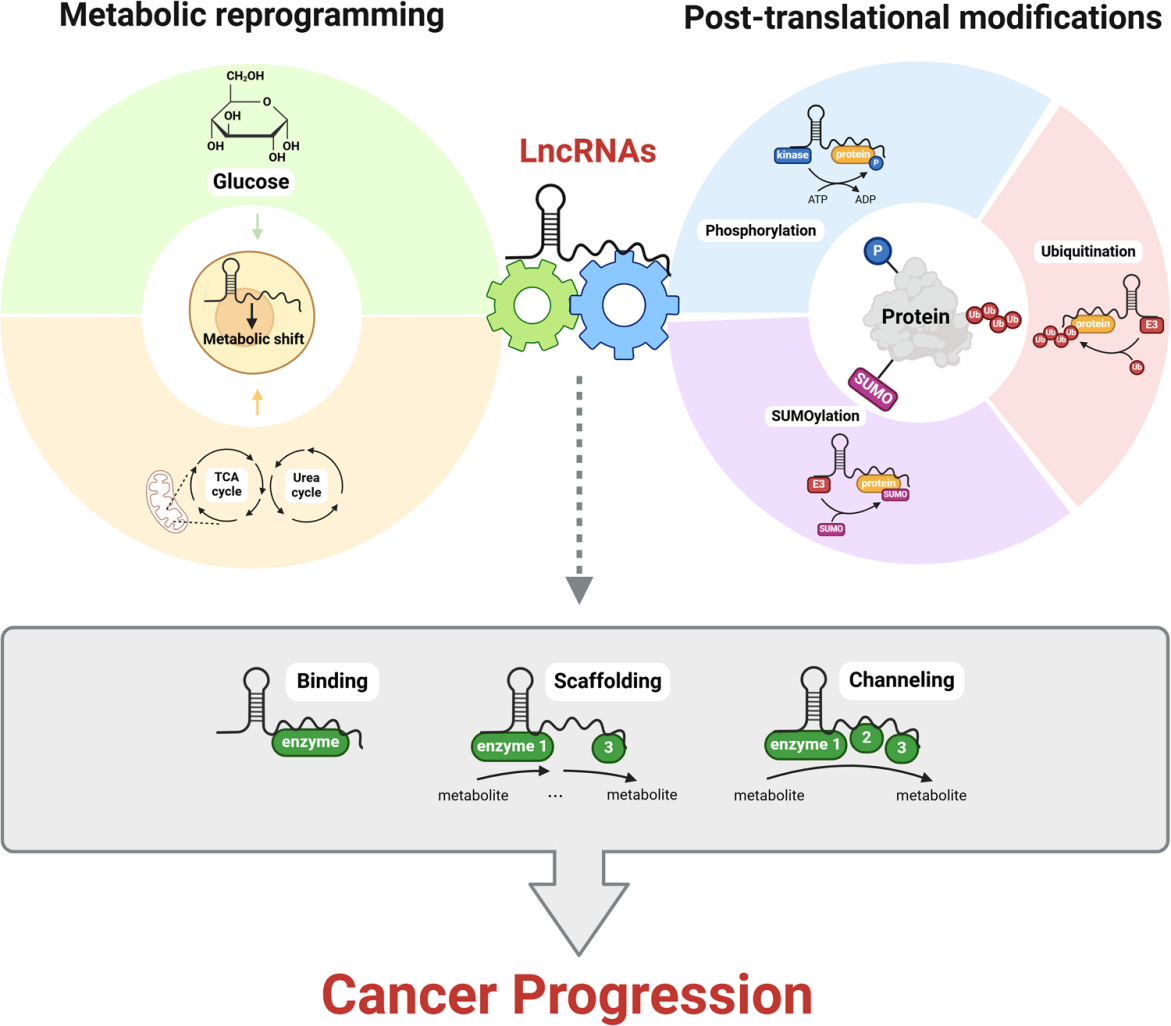
LncRNAs serve as regulators that fine-tune metabolic reprogramming and PTMs. LncRNAs influence cancer progression by binding to metabolic enzymes and regulating their activity. Enzymes within the same metabolic pathway can interact with lncRNA scaffolds. In addition, lncRNAs can modulate various protein modifications by enhancing the efficiency of protein modifications such as phosphorylation, ubiquitination, and SUMOylation. Through these activities, lncRNAs serve as fine-tuning regulators of key PTMs that influence cancer progression.

In this Review, we highlight the role of lncRNAs in cancer through their direct interactions with proteins, categorizing their functions into the following primary mechanisms, which are discussed in greater detail: (1) modulating the activity and proximity of metabolic enzymes and (2) regulating enzymatic processes associated with PTMs, ultimately influencing cancer development. Together, these findings suggest that lncRNAs are not merely ‘noncoding RNAs’ but function as fine-tuning regulators in cancer, offering deeper insights into the modulation of cellular processes such as cancer metabolism and PTMs, while providing new perspectives on cancer pathogenesis and treatment (Fig. 1).

## LncRNAs as modulators of metabolic enzyme activity

Research into the modulation of cancer metabolism remains in its early stages, leading to a limited understanding of this complex process. Therefore, in this section of the Review, we aim to provide comprehensive insights into lncRNAs as critical regulators of metabolic enzymes and how variations in enzyme expression or activity impact cancer reprogramming. Specifically, lncRNAs can regulate metabolic enzymes by forming complexes with them. Recent studies demonstrate that lncRNAs interact with these enzymes either by directly binding to them or by serving as scaffolds and coordinating multiple enzymes within the same metabolic pathway to enhance metabolic flux. The relationships between lncRNAs and metabolic enzymes discussed in this section are summarized in Table 1.

**Table 1:** LncRNAs regulating energy metabolism by binding with metabolic enzymes.

Related metabolism	LncRNAs	Proteins or enzymes	Methods to study binding with lncRNAs	Molecular mechanism	Cancer phenotypes	Cancer types	Reference
Glycolysis	*lncRNA-6195*	ENO1	RNA-affinity-pull-down–MS, RIP	Inhibits ENO1 enzymatic activity	Inhibits cell proliferation in vitro and HCC growth in vivo	Hepatocellular cancer	^43^
	*NEAT1_1*	PGK1, PGAM1, and ENO1	In vitro RNA pull-down with recombinant PGK1, PGAM1 and ENO1 proteins, fluorescence polarization assay, cross-linking IP-qPCR	Accelerates glycolysis by substrate channeling of PGK1, PGAM1, and ENO1	Promotes cell proliferation and metastasis	Breast cancer	^44^
	*gLINC*	PGK1, PGAM1, ENO1, PKM2 and LDHA	RNA-affinity pull-down, sucrose density gradient centrifugation, IF, co-IP, fluorescence in situ hybridization (FISH), RIP	Enhances glycolytic flux by channeling of PGK1, PGAM1, ENO1, PKM2, and LDHA	Promotes cell proliferation in vitro and tumor growth in vivo	Hepatocellular cancer, CRC and cervical cancer	^45^
	*HULC*	LDHA and PKM2	Surface plasmon resonance, TOBAP-MS	Enhances phosphorylation of both LDHA, and PKM2, then increases glycolysis	Promotes cell proliferation in vitro	Hepatocellular cancer	^46^
	*WFDC21P*	PFKP and PKM2	RIP, IP using MS2-tagged RNA aptamer	Disrupts the PFKP tetramer formation and inhibits of PKM2 nuclear translocation	Inhibits cell proliferation, metastasis in vitro and tumor growth in vivo	Hepatocellular cancer	^47^
TCA cycle	*TINCR*	ACLY	RNA-affinity-pull-down–MS, RNA-EMSA	Protects from ubiquitination and maintains concentration of acetyl-CoA	Promotes cell proliferation, migration and invasion in vitro and tumor growth in vivo	Nasopharyngeal cancer	^53^
	*IDH1-AS1*	IDH1	RNA-affinity pull-down, RIP	Enhances the enzymatic activity by dimerization of IDH1	Inhibits cell proliferation in vitro and tumor growth in vivo	Cervical cancer	^54^
	*GAS5*	MDH2 and CS	RNA-affinity-pull-down–MS, co-IP	Disrupts FH, MDH2, and CS interactions	Inhibits cell proliferation in vitro and tumor growth in vivo	Breast cancer	^55^
	*AC020978*	MDH2	RNA-affinity-pull-down–MS, RIP	Protects from ubiquitination and activates the AKT signaling pathway by binding with MDH2	Promotes cell migration and invasion in vitro and metastasis in vivo	NSCLC	^56^
Arginine	*LOC113230*	ASS1	RNA-affinity-pull-down–MS, RIP, co-IP	Facilitates assembly of LRPPRC–TRAF2 E3 complex and induces ubiquitination on K234 residue	Inhibits cell proliferation and migration in vitro and tumor growth in vivo	CRC	^58^

### LncRNA-mediated regulation of glycolysis

Mammalian cells reprogram cellular metabolism when they require additional energy and/or macromolecular building blocks to accelerate processes such as cellular division, proliferation, quiescence, senescence, differentiation, tumorigenesis or metastasis^4,32,33^. Glucose plays a critical role in cancer metabolism, serving as the primary energy source for rapidly proliferating cancer cells. Relative to normal cells, cancer cells require more energy and macromolecular building blocks for efficient glucose utilization and nucleoside and nucleotide synthesis^7^. A well-known example of these cancer-specific metabolic changes is the Warburg effect, wherein tumor cells increase glucose uptake and enhance anaerobic glycolysis, producing lactate and accumulating energy under hypoxic conditions in a manner that permanently alters cellular characteristics^34–38^.

LncRNAs can influence glycolysis through interactions with enzymes. The ability of certain metabolic enzymes to bind RNAs has been shown to play a significant role in maintaining metabolic and physiological homeostasis in specific contexts^39,40^. Enhanced cross-linking and immunoprecipitation (IP) studies have revealed that enolase 1 (ENO1), a critical enzyme in glycolysis, binds not only to cellular mRNAs but also to lncRNAs^41–43^. Yu et al. demonstrated that *lncRNA-6195* is part of a complex containing ENO1, inhibiting ENO1’s enzymatic activity, glucose consumption, and lactate abundance in hepatocellular carcinoma (HCC). To identify ENO1 binding sites on *lncRNA-6195*, a series of *lncRNA-6195* deletion mutants were constructed and an RNA pull-down assay was performed. The results revealed that nucleotides 101 and 529 of *lncRNA-6195* are responsible for forming the complex with ENO1. In addition, Flag-tagged deletion mutants of ENO1 were generated, and an RNA immunoprecipitation (RIP) assay was conducted to map the *lncRNA-6195*-binding domain on ENO1. Western blotting analyses of Flag-tagged ENO1 and its mutants revealed that *lncRNA-6195* binds to the 237–405 amino acid region of ENO1. The loss of ENO1 enzymatic activity due to mutations in the substrate-binding site was confirmed by reduced levels of phosphoenolpyruvate under conditions of *lncRNA-6195* overexpression^43^. This suggests that a metabolic shift from glycolysis to oxidative phosphorylation may occur under these conditions. Identifying a specific lncRNA capable of preferentially binding to ENO1 over other RNA-binding proteins could be valuable, given the active roles that lncRNAs play in regulating aerobic glycolysis and other metabolic pathways in cancer.

Furthermore, some lncRNAs influence metabolic processes by serving as scaffolds for enzymes involved in the same metabolic pathway to increase metabolic flux efficiency. The short isoform of NEAT1 (*NEAT1_1*) plays a crucial role in promoting aerobic glycolysis in breast cancer. *NEAT1_1* accelerates glycolytic activity in breast cancer through substrate channeling of PGK1, PGAM1, and ENO1, bringing them into close association in the cytoplasm. This association has been confirmed through IP after incubating recombinant PGK1, PGAM1 and ENO1 proteins with in vitro-transcribed sense or antisense *NEAT1_1*, as well as fluorescence polarization assays. The scaffolding of these enzymes mediated by *NEAT1_1* facilitates the penultimate step in glycolysis, allowing breast cancer cells to consume glucose more rapidly. Consistently, mammary tumor weight and size were reduced in Neat1^−/−^ mice compared with Neat1^+/+^ mice crossed with MMTV-PyVT transgenic mice. *NEAT1_1* thus accelerates glycolysis in breast cancer, promoting cancer progression and metastasis and contributing to a poorer prognosis for patients^44^.

Similar to NEAT1 in breast cancer, the c-Myc-responsive lncRNA glycoLINC (*gLINC*) promotes glycolysis by acting as a scaffold for glycolytic enzymes, including PGK1, ENO1, PKM2, and LDHA, with indirect binding to PGAM1. This was revealed through co-IP results from cell lysates in which short hairpin RNA (shRNA) against *gLINC* was introduced, showing a decrease in the assembly of complexes containing PGK1, PGAM1, ENO1, and LDHA. Under conditions of serine and glycine starvation, *gLINC* enhances glycolytic flux and accelerates ATP production to replenish energy reserves, thereby sustaining tumor cell viability. Using affinity *gLINC* pull-down and RIP approaches, researchers demonstrated the ability of *gLINC* to bind these four glycolytic enzymes and LDHA simultaneously. In addition, immunofluorescence (IF) and fluorescence in situ hybridization (FISH) suggested the potential co-localization of *gLINC* with these enzymes, although it remains unclear whether they form a biochemical complex together at the same time and place. Furthermore, sucrose density gradient centrifugation demonstrated that the PGK1, PGAM1, ENO1, and LDHA complexes were found in the same fraction, which was absent under *gLINC* knockdown conditions, highlighting its potential role in mediating substrate channeling^45^.

Furthermore, the lncRNA *HULC* (highly upregulated in liver cancer) directly binds to LDHA, and PKM2, as demonstrated via surface plasmon resonance using recombinant LDHA and in vitro-transcribed *HULC*. The dissociation constant (*K*_D_) was 2.898 × 10^−8^ M for the LDH enzyme. Moreover, tobramycin affinity purification and mass spectrometric analysis (TOBAP-MS) confirmed the interaction between *HULC*, LDHA and PKM2, revealing the nature of their binding. Mechanistically, *HULC* acts as an adaptor, enhancing their interaction with the intracellular domain of fibroblast growth factor receptor type 1 (FGFR1). This mechanism enhances the phosphorylation of both LDHA and PKM2, thereby accelerating aerobic glycolysis, as measured by the extracellular acidification rate, glucose uptake, lactate abundance, and acetyl-CoA concentration, ultimately promoting cellular proliferation in HepG2 cells^46^.

**Fig. 2:**
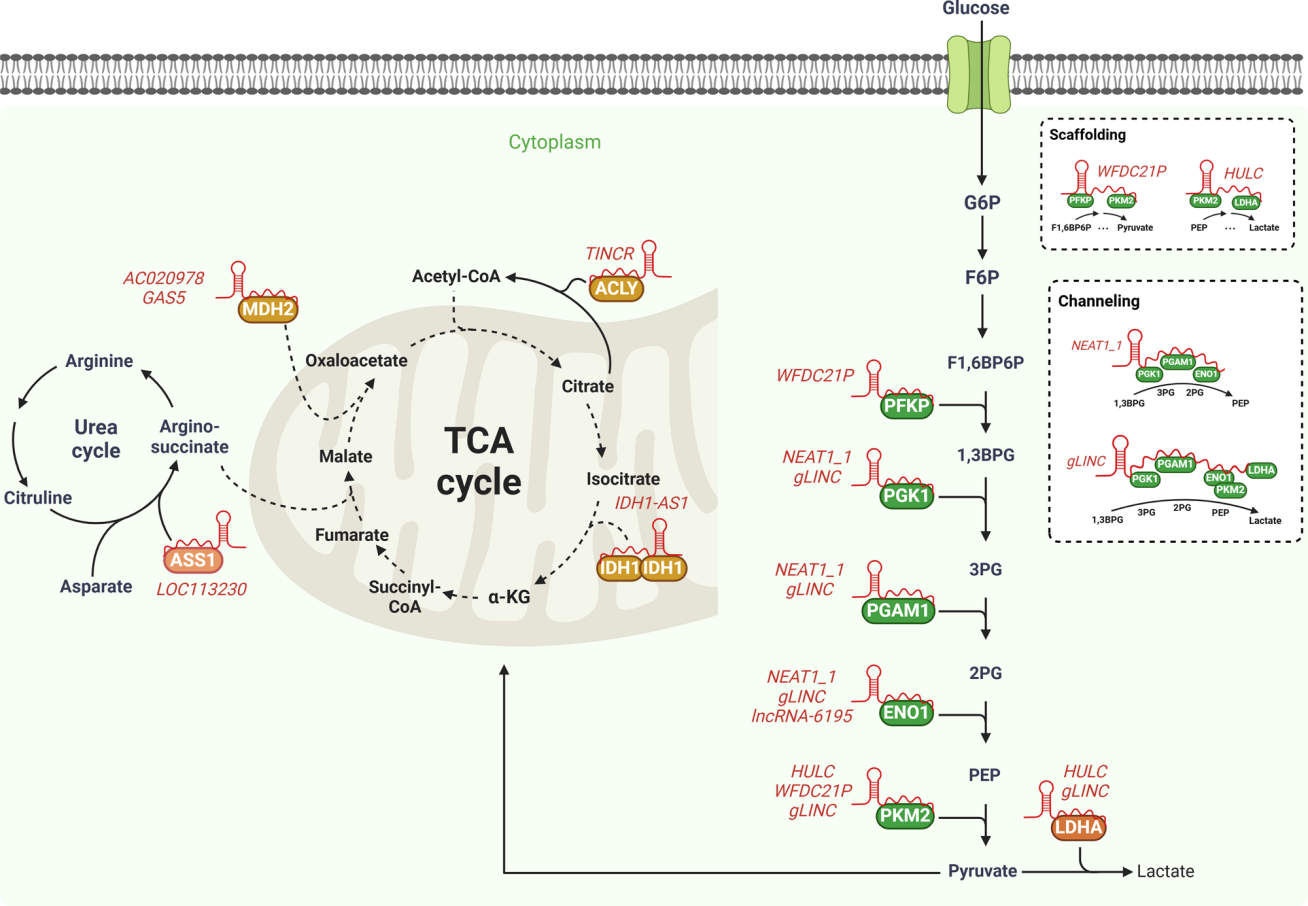
LncRNAs directly bind enzymes to regulate their activity. LncRNAs bind to metabolic enzymes and alter their activity. Glycolysis generates pyruvate, which then enters the mitochondrial TCA cycle to produce ATP through oxidative phosphorylation through a series of enzymatic reactions. LncRNAs including *WFDC21P*, *HULC*, *NEAT1_1,* and *gLINC* can accelerate glucose metabolism through scaffolding and channeling mechanisms. In addition, lncRNAs including *TINCR*, *IDH1-AS1,* and *AC020978* bind to specific metabolic enzymes in the TCA cycle, enhancing associated metabolic activity. By contrast, lncRNAs such as *lncRNA-6195*, *GAS5,* and *LOC113230* inhibit the activity of individual metabolic enzymes. Collectively, these findings suggest that lncRNAs impact ATP production, emphasizing their importance in the metabolic reprogramming of cancer.

The orphan nuclear receptor Nur77 increases the abundance of the lncRNA *WFDC21P*, which binds to PFKP and PKM2 to inhibit glycolysis by reducing the extracellular acidification rate, glucose uptake, and lactate abundance in HCC. IP analyses using the MS2 RNA aptamer system and RIP assays revealed that *WFDC21P* strongly binds to PFKP and subsequently inhibits the tetramer formation necessary for PKM2 nuclear translocation. By disrupting the functions of these two key glycolytic enzymes, *WFDC21P* acts as an inhibitor of HCC cell viability and tumorigenesis^47^ (Fig. 2).

### LncRNA-mediated regulation of the TCA cycle

The tricarboxylic acid (TCA) cycle is a central hub for cellular energy metabolism, redox balance, and macromolecule biosynthesis. It plays a pivotal role in tumorigenesis by enabling cancer cells to efficiently meet their biochemical demands using diverse biofuels such as fatty acids, amino acids, and glucose^48,49^. Recent discoveries have highlighted the critical involvement of the TCA cycle and its products in cancer-associated metabolic reprogramming, emphasizing its potential as a therapeutic target despite the Warburg effect^50,51^. Moreover, lncRNAs have emerged as key regulators of this metabolic pathway, physically binding to and modulating various TCA cycle enzymes, thereby influencing tumor initiation, development, and progression. ATP is essential for nearly all cellular activities, making its efficient acquisition crucial for cell survival. The majority of ATP is produced via oxidative phosphorylation in mitochondria, driven by the TCA cycle. This process remains an important energy source even in cancer cells with active glucose–lactate fermentation^48,52^.

The lncRNA *TINCR* is overexpressed in nasopharyngeal carcinoma and interacts with ATP citrate lyase (ACLY), an enzyme that metabolizes citrate to acetyl-CoA. This interaction protects ACLY from ubiquitin-mediated degradation, thereby maintaining intracellular acetyl-CoA levels. The interaction between ACLY and *TINCR* was identified through a *TINCR* pull-down assay combined with mass spectrometry (MS), while an RNA electrophoretic mobility shift assay (RNA-EMSA) further validated the direct interaction between biotin-labeled *TINCR* and recombinant ACLY^53^.

Furthermore, the lncRNA *IDH1-AS1* enhances the enzymatic activity of IDH1, a key enzyme in the TCA cycle that converts isocitrate to α-ketoglutarate (α-KG) under normoxic conditions. *IDH1-AS1* facilitates IDH1 dimerization by binding to it, as confirmed through RNA-affinity pull-down, RIP, and IP assays using truncation mutants of IDH1 and *IDH1-AS1* in HeLa cell lysates. This binding increases α-KG levels, boosting TCA cycle activity, thereby reducing reactive oxygen species levels and downregulating HIF1α, ultimately suppressing glycolysis. However, *IDH1-AS1* transcription is repressed by c-Myc, which prevents TCA cycle enhancement, sustaining HIF1α and perpetuating the Warburg effect under normoxic conditions. This promotes glycolysis, as indicated by elevated glucose and lactate levels, thereby enhancing cancer cell clonogenicity and tumor growth^54^.

Moreover, Sang et al. identified *GAS5* as a mitochondria-localized lncRNA that functions as a tumor suppressor in breast cancer by directly binding to target enzymes, highlighting its essential role in maintaining cellular energy homeostasis and inhibiting tumor growth. *GAS5* exerts its function in response to energy stress by disrupting metabolic interactions among key TCA cycle enzymes, including fumarate hydratase (FH), malate dehydrogenase 2 (MDH2), and citrate synthase (CS), thereby modulating mitochondrial TCA flux. Elevated *GAS5* levels correlate with reduced TCA flux, which is associated with favorable clinical outcomes in patients with breast cancer. These findings highlight the role of *GAS5* in regulating metabolic pathways critical to cancer progression and suggest its potential as a prognostic biomarker and therapeutic target, underscoring the unexplored but significant role of mitochondria-related lncRNAs in cancer onset and progression^55^.

Furthermore, Xu et al. identified the lncRNA *AC020978* as a key regulator of MDH2, facilitating non-small cell lung cancer (NSCLC) progression. RNA-affinity pull-down assays followed by MS, along with RIP assays using full-length and truncated MDH2 constructs in A549 and H1299 cell lysates, confirmed the interaction between *AC020978* and MDH2. This interaction enhances MDH2 stability and functionality by protecting it from ubiquitin-mediated proteasomal degradation, leading to increased conversion of α-KG to 2- hydroxyglutarate (2-HG). Consequently, AC020978 activates the AKT signaling pathway and promotes metastatic phenotypes in NSCLC cells. Clinical FISH analysis of tissues from patients with NSCLC demonstrated that *AC020978* is overexpressed in metastatic NSCLC compared with nonmetastatic cases. Moreover, survival analysis revealed that elevated *AC020978* levels correlate with poor overall survival and recurrence-free survival in patients with NSCLC. Consequently, *AC020978* may serve as a potential biomarker and therapeutic target in metastatic NSCLC. These findings underscore the significance of lncRNA interactions with TCA cycle enzymes in cancer metabolism^56^ (Fig. 2).

### LncRNA-mediated regulation of arginine metabolism

Arginine is synthesized through a two-step process from citrulline. Argininosuccinate synthase 1 (ASS1) converts citrulline to argininosuccinate, which is then converted to arginine by argininosuccinate lyase (ASL)^57^. In the context of arginine metabolism in colorectal cancer (CRC) cells, the lncRNA *LOC113230* functions as a scaffold for leucine-rich pentatricopeptide repeat-containing protein (LRPPRC) and TNF receptor-associated factor 2 (TRAF2), both of which are E3 ubiquitin ligases. This interaction enhances the binding of these proteins to ASS1, facilitating the formation of the LRPPRC–TRAF2 complex. This complex assembly, mediated by *LOC113230*, promotes the degradation of ASS1 through K234 ubiquitination, as identified by tandem MS and confirmed by IP followed by western-blotting-mediated ubiquitin detection. This degradation reduces intracellular arginine levels, thereby inhibiting the metastasis and growth of CRC cells^58^ (Fig. 2).

## LncRNAs as regulators of protein modifications

In addition to modulating metabolic enzymes, lncRNAs are also implicated in the regulation of enzymatic reactions crucial for cellular functions. Among these, PTMs play a pivotal role in regulating protein function, stability, and localization^59^. As fine-tuning regulators, lncRNAs influence PTMs, thereby altering signaling pathways and cellular responses, ultimately contributing to the complexity of cancer pathogenesis^60^. In this section of the Review, we highlight additional perspectives on the interactions between lncRNAs and enzymes involved in PTMs in cancer, as summarized in Table 2.

**Table 2:** LncRNAs binding with enzymes of protein modifications.

Protein modifications	LncRNAs	Proteins or enzymes	Methods to study binding with lncRNAs	Molecular mechanisms	Cancer phenotypes	Cancer types	References
Phosphorylation	*LINP1*	eIF2α	RNA-affinity pull-down–MS, RIP	Suppresses phosphorylation catalyzed by the PERK	Promotes cell proliferation, colony formation, migration and invasion in vitro and tumor growth in vivo	Cutaneous squamous cancer	^74^
	*CamK-A*	PNCK	RNA-affinity pull-down–MS, in vitro RNA pull-down with recombinant PNCK and IκBα, RIP, in vitro kinase assays	Binds and promotes the phosphorylation of IκBα	Promotes cell proliferation, invasion, metabolic adaptation in vitro	Breast cancer	^60^
Ubiquitination	*HOTAIR*	DZIP3-Ataxin-1 and MEX3B-SNP	RIP, in vitro RNA pull-down	Interacts with substrates Ataxin-1 or Snurportin and degrades the proteins rapidly	Promotes cellular senescence	Fibroblast, cervical cancer	^79^
SUMOylation	*PSTAR*	hnRNP K	RNA-affinity pull-down–MS, RIP, co-IP, luciferase reporter assays	Protects ubiquitination of p53	Inhibits cell proliferation in vitro	Hepatocellular cancer	^88^

### Protein phosphorylation

Protein phosphorylation is a major PTM that functions as a molecular switch to regulate various cellular processes, including cell signaling, growth, differentiation, and death^19^. This modification alters protein localization, activity, degradation and interactions with other proteins by adding phosphoryl groups, primarily to serine, threonine, and tyrosine residues^61^. It is catalyzed by protein kinases, which themselves are regulated through intricate signaling networks^62,63^. Protein phosphorylation enables the rapid transmission of intracellular signals to help cells adapt to environmental stress. Phosphorylation of eukaryotic initiation factor 2α (eIF2α) is crucial for cancer adaptation to stress and is primarily regulated by four phosphorylation kinases^64,65^. Protein kinase R (PKR) is activated by abnormal concentrations of double-stranded RNAs, such as those from viral infections, leading to eIF2α phosphorylation and subsequent inhibition of protein synthesis^66,67^. Similarly, PKR-like endoplasmic reticulum kinase (PERK) responds to endoplasmic reticulum (ER) stress by phosphorylating eIF2α, playing a key role in cellular stress responses by regulating protein synthesis^68^. In addition, the heme-regulated eIF2α kinase (HRI), activated by heme deficiency, and the general control nonderepressible 2 (GCN2) pathway, activated by nutrient deprivation, independently phosphorylate eIF2α^69,70^. This phosphorylation inhibits global translation while enhancing the selective translation of activating transcription factor 4 (ATF4), a master regulator that governs key adaptive gene transcription^71^. This process, known as the integrated stress response, is essential for maintaining cellular homeostasis^72,73^.

Recent research indicates that lncRNAs can inhibit eIF2α phosphorylation in cancer. The lncRNA *LINP1* is significantly upregulated in cutaneous squamous cell carcinoma (cSCC) and is potentially involved in the ER stress response. RNA sequencing of siLINP1-transfected cSCC cells revealed gene expression changes, and *LINP1* was found to physically interact with eIF2α. This interaction was demonstrated through a *LINP1* pull-down assay followed by MS using cSCC cell lysates and further validated by RIP and IF assays. The interaction inhibits PERK binding to eIF2α, as shown through co-IP and western blotting analyses in *LINP1*-overexpressing cells, ultimately reducing eIF2α phosphorylation. This mechanism suppresses the ER stress response, inhibits apoptosis, and promotes cell proliferation, colony formation, migration, and invasion, enhancing cSCC survival under ER stress conditions^74^.

**Fig. 3:**
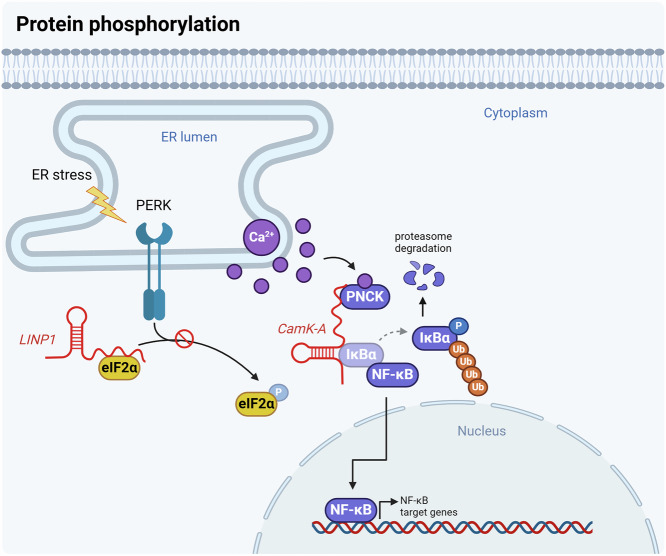
LncRNAs interact with various enzymes to regulate protein phosphorylation. PTMs directly impact protein function. Protein phosphorylation, in particular, is essential for intracellular signaling pathways, and lncRNAs such as *LINP1* can modulate phosphorylation dynamics. Notably, *CamK-A* plays a crucial role in the NF-κB pathway by binding to PNCK. These PTMs significantly affect cellular processes.

Moreover, Sang et al. identified the lncRNA *CamK-A*, which facilitates cell viability and glucose uptake in MDA-MB-231 cells through small interfering RNA (siRNA) library-based screening. They found that *CamK-A* silencing significantly inhibited NF-κB transcriptional activity in a pathway reporter array. *CamK-A* pull-down and MS analyses revealed that this lncRNA binds to calcium/calmodulin-dependent protein kinase (PNCK) and IκBα, with these interactions having been validated through RIP and RNA-protein binding assays. The study also suggested that interaction with *CamK-A* promotes conformational changes in PNCK, enhancing its kinase activity. IκBα is another *CamK-A* binding partner. In vitro kinase assays revealed that PNCK phosphorylates IκBα at Ser32. Under hypoxic conditions, elevated cytosolic calcium concentrations facilitate IκBα phosphorylation by both *CamK-A* and PNCK, thereby promoting nuclear NF-κB p65 translocation through the degradation of IκBα. Consequently, the nuclear accumulation of NF-κB p65, facilitated by *CamK-A*–PNCK–IκBα complexes, induces the transcription of NF-κB target genes, promoting cancer progression through the remodeling of the tumor microenvironment^60^ (Fig. 3).

These findings collectively suggest that lncRNAs regulate protein phosphorylation, influencing cancer growth and metastasis. However, the regulatory roles of lncRNAs in phosphorylation remain insufficiently understood, and more diverse mechanisms are likely to be uncovered. In particular, interactions between lncRNAs and key phosphorylation enzymes such as HRI and GCN2 in conserved integrated stress responses remain unexplored. Further studies are needed to elucidate how lncRNAs regulate phosphorylation across various cancer types.

### Protein ubiquitination

Protein ubiquitination is mediated by a series of catalytic enzymes that conjugate monomeric or polymeric ubiquitin to target proteins. This process significantly influences cell survival, differentiation, and death by regulating protein degradation^75,76^. Ubiquitination occurs through three sequential steps catalyzed by ubiquitin-activating enzymes (E1), ubiquitin-conjugating enzymes (E2) and ubiquitin protein ligases (E3)^77,78^. Mono-ubiquitin proteins bind to lysine residues on target proteins and form poly-ubiquitin chains, leading to protein degradation by the 26S proteasome. Recent studies have revealed that lncRNAs can act as regulators of protein ubiquitination.

**Fig. 4:**
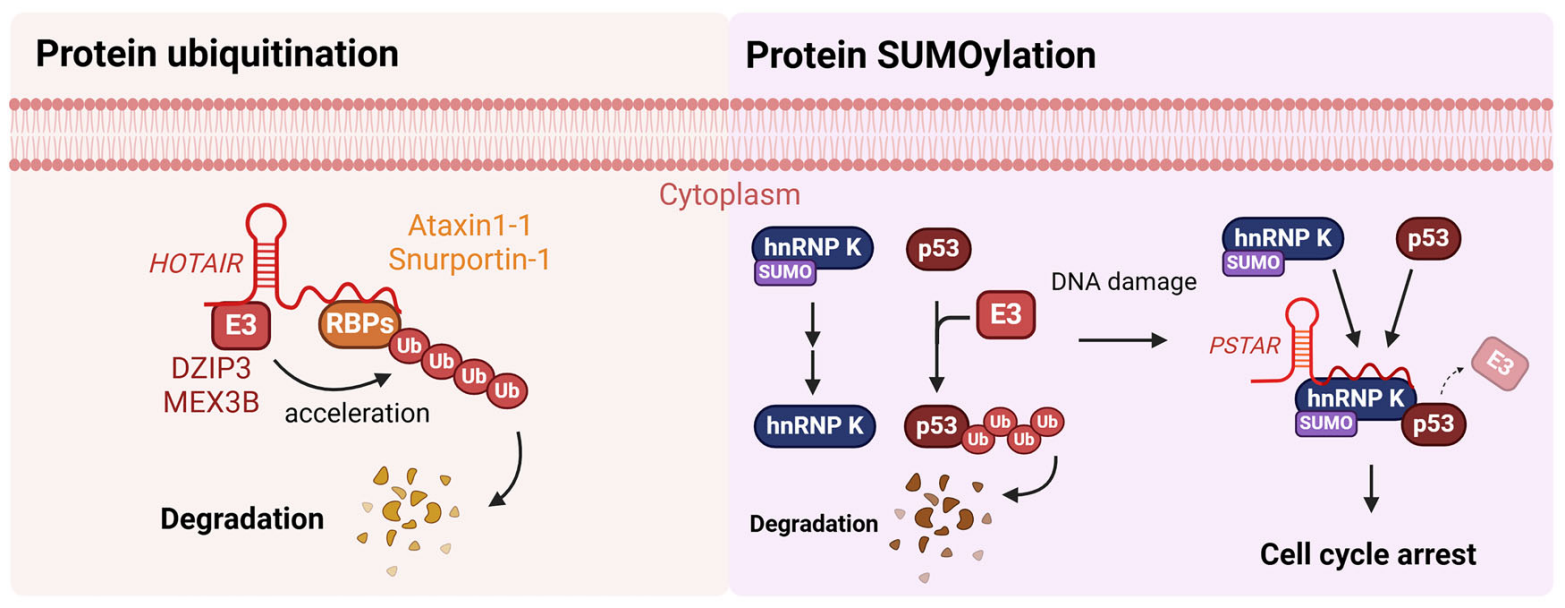
LncRNAs regulate protein ubiquitination and SUMOylation. Protein ubiquitination and SUMOylation are critical PTMs that govern protein degradation. The lncRNA *HOTAIR* interacts with E3 ubiquitin ligases and their substrates to regulate the stability of target proteins. Similarly, the lncRNA *PSTAR* binds to SUMOylation-related proteins to control the proteasomal degradation of target proteins. These lncRNAs can influence cancer progression through protein modifications.

Yoon et al. demonstrated that the lncRNA *HOTAIR* functions as a scaffold that enhances the activity of a complex containing the E3 ubiquitin ligases DZIP3 and MEX3B by directly binding to these ligases and their substrates, Ataxin-1 and Snurportin-1. RIP analysis using HeLa cell lysates confirmed the interactions of *HOTAIR* with these proteins. Specifically, RIP analysis with RNase T1 treatment and RT-qPCR (Reverse Transcription Quantitative PCR) experiments, which divided *HOTAIR* RNA into 23 fragments, identified the specific binding regions for each protein. DZIP3 predominantly binds to fragment 12 (nucleotides ~1,028 to 1,272), Ataxin-1 interacts with fragments 11 and 12, MEX3B binds to fragment 2 and Snurportin-1 binds to fragment 12. In addition, in vitro ubiquitination experiments demonstrated that *HOTAIR* significantly enhances the ubiquitination of Ataxin-1 and Snurportin-1 with the assistance of E2 enzymes UbcH6 and UbcH10. In systems containing E1 and E2 enzymes, along with the E3 ligases DZIP3 and MEX3B, *HOTAIR* was shown to promote the ubiquitination of target proteins, leading to their rapid degradation via the proteasome. This underscores the role of *HOTAIR* in increasing the proximity between E3 ligases and their substrates, thereby enhancing ubiquitination efficiency. The function of *HOTAIR* in ubiquitin-mediated protein degradation has also been linked to the induction of cellular senescence, during which *HOTAIR* is notably overexpressed^79^. While senescence acts as a barrier to tumor formation by halting cell proliferation, the accumulation of senescent cells creates a pro-inflammatory microenvironment that can promote tumor progression^80^. The *HOTAIR*-induced senescence highlights its paradoxical role in shaping a tumor-supportive environment, emphasizing its potential as a regulator in cancer progression^81^ (Fig. 4).

### Protein SUMOylation

SUMOylation is a PTM in which a small ubiquitin-like modifier (SUMO) is attached to specific lysine residues of a protein^82,83^. This modification plays a crucial role in regulating protein activity and localization, affecting various cellular processes such as genome stability and mRNA processing^84,85^. Notably, SUMOylation modulates several aspects of cancer progression, including proliferation, tumor formation, transformation, and invasion^86^. Although the SUMO protein shares only 18% sequence identity with ubiquitin, its three-dimensional structural similarity—including the ββαββαβ fold and two C-terminal glycine residues—enables it to influence protein degradation^87^.

Qin et al. identified a lncRNA termed p53-stabilizing and activating RNA (*PSTAR*), which plays a significant role in suppressing HCC. Through integrative omics analysis, they found that *PSTAR* inhibits HCC cell proliferation and tumorigenicity by inducing p53-mediated cell cycle arrest. Mechanistically, PSTAR binds to heterogeneous nuclear ribonucleoprotein K (hnRNP K), as demonstrated by RNA-affinity pull-down assays followed by MS, as well as RIP assays. This interaction enhances the association between hnRNP K and p53, leading to increased p53 expression levels, as shown by western blotting, and enhanced p53 activity, as demonstrated using luciferase reporter assays. Furthermore, *PSTAR* inhibits SENP2-mediated deSUMOylation of hnRNP K, as confirmed through co-IP experiments with hnRNP K and SENP2, along with IP followed by western blotting under conditions of SENP2 silencing. This inhibition promotes the SUMOylation of hnRNP K, strengthening its interaction with p53. Consequently, this interaction prevents MDM2-mediated ubiquitination and degradation of p53, thereby increasing its stability. This was validated by assessing p53 protein stability under cycloheximide treatment to inhibit new protein synthesis. Ultimately, *PSTAR* activates p53 target genes such as p21 and SFN, leading to cell cycle arrest and inhibition of tumor proliferation^88^ (Fig. 4).

## Concluding remarks

Advances in next-generation sequencing have revealed that aberrant lncRNA expression is associated with various cancer types. However, compared with the total number of lncRNAs believed to have biological functions, only a small fraction have been well characterized. This Review summarizes current knowledge regarding the diverse mechanisms by which lncRNAs regulate the activity of enzymes involved in metabolic reprogramming and PTMs in cancer. Through diverse mechanisms, lncRNAs act as fine-tuning regulators, playing a critical role in cancer development and progression. In the field of metabolic reprogramming, much research has focused on glycolysis. However, as glucose alone is insufficient to sustain life, broader studies on the metabolism of other amino acids are needed. In addition, while PTMs are crucial modifications that can directly alter enzyme function, research on their association with lncRNAs remains limited. Understanding these complex regulatory networks provides valuable insights into cancer mechanisms and highlights the potential of lncRNAs as therapeutic targets. However, when investigating the biological roles of lncRNAs, it is essential to acknowledge that many proteins and enzymes identified as direct binding partners may, in reality, interact indirectly. This is because RIP and IP experiments do not directly demonstrate interactions between lncRNAs and target molecules but rather show their co-existence within a complex. Thus, the possibility of intermediary factors mediating these interactions underscores the need for a more rigorous approach in lncRNA research. Future studies exploring the intricate interactions between lncRNAs and specific enzymatic pathways will be pivotal in developing novel cancer therapies.
